# Bi-allelic *EPHA2* mutations cause dysosteosclerosis by inhibiting osteoclastogenesis and enhancing osteoblastogenesis

**DOI:** 10.1038/s41413-026-00565-x

**Published:** 2026-10-12

**Authors:** Xi Chen, Xin Li, Zenghui Gu, Qi Lu, Xinyu Qian, Yier Fan, Ke Deng, Shanshan Li, Zhe Wei, Yang Xu, Wenzhen Fu, Hou-Feng Zheng, Weiwei Hu, Zhenlin Zhang

**Affiliations:** 1 Shanghai Clinical Research Center of Bone Diseases, Department of Osteoporosis and Bone Diseases, Shanghai Sixth People’s Hospital Affiliated to Shanghai Jiao Tong University School of Medicine, Shanghai, China; 2 Department of Endocrinology and Metabolism, Shanghai Children’s Medical Center, School of Medicine, Shanghai Jiao Tong University, Shanghai, China; 3 Suzhou Laboratory of Precision Health and Data Science, the Second Affiliated Hospital of Soochow University, Suzhou, Jiangsu China; 4 Institute of Health Data Science, Soochow University, Suzhou, Jiangsu China; 5 Institute of Basic Medical Sciences, Westlake Institute for Advanced Study and Westlake University, Hangzhou, China; 6 Clinical Research Center, Shanghai Sixth People’s Hospital Affiliated to Shanghai Jiao Tong University School of Medicine, Shanghai, China

**Keywords:** Osteopetrosis, Calcium and phosphate metabolic disorders, Osteoporosis

## Abstract

Erythropoietin-producing hepatocellular (EPH) receptors, the largest receptor tyrosine kinase family, have been shown to play a crucial role in normal physiological processes and pathologies. EPHA2, encoded by *EPHA2* gene, a member of the EPH receptor family, has been implicated in cataracts and various bone tumors, including osteosarcoma, chondrosarcoma, and bone metastases of breast cancer. However, no association between the *EPHA2* gene and genetic skeletal disorders has been reported to date. Here, we identified a Chinese family with a diagnosis of dysosteosclerosis, presenting with significant increase in lumbar spine bone mineral density, multifocal sclerotic lesions throughout the body, sclerotic skull, platyspondyly and sandwich vertebrae, and demonstrated compound heterozygous mutations in *EPHA2* gene (c.890 C > T, p.Thr297Met; c.988dupT, p.Ser330PhefsX51). We observed a reduction in numerous sclerotic lesions in patients during the five-year follow-up. To systematically investigate the molecular mechanisms of pathogenesis, we employed a range of methodologies, including in vitro cellular assays, genome-wide association studies analysis, induced pluripotent stem cells models and *Epha2* knockout mice models. We have determined that loss-of-function mutations in *EPHA2* gene lead to dysosteosclerosis through enhancing mesenchymal stem cells osteogenic differentiation and downregulating the p38-MAPK pathway, leading to decreased osteoclast differentiation. Consequently, we proposed and validated *EPHA2* as a viable therapeutic target for osteoporosis by constructing femoral fracture mice models and ovariectomized mice models. In summary, this study identifies bi-allelic loss-of-function mutations in *EPHA2* gene lead to dysosteosclerosis and its underlying mechanisms, and suggests *EPHA2* as a potential therapeutic target for osteoporosis.

## Introduction

Bone mass and strength are maintained by the balance between osteoclast-mediated bone resorption and osteoblast-mediated bone formation throughout life.^1,2^ Sclerosing bone dysplasias are rare monogenic diseases characterized by a significantly increased bone mass with a wide range of radiologic, clinical, and genetic characteristics.^3–5^ In the latest report of the “Nosology of genetic skeletal disorders: 2023 revision”, the diseases with an increased bone mass are subdivided in two categories: Osteopetrosis and related osteoclast disorders (Group 24), which include osteopetrosis (OMIM 166600) and dysosteosclerosis (DOS; OMIM 224300) and are characterized by impaired osteoclast development or function, and Osteosclerotic disorders (Group 25).^6^

DOS is a rare autosomal recessive sclerosing bone dysplasia characterized by osteosclerosis and platyspondyly.^7^ Irregular osteosclerosis, flattened diffusely dense vertebral bodies, sclerotic skull, radiolucent submetaphyseal portions of the long tubular bones with sclerotic diaphysis are radiological features of DOS.^8^ Exclusion criteria for DOS include the presence of diffuse osteosclerosis and persistent bone marrow medullary space occupation.^9^ Following extended follow-up, patients with DOS demonstrated the reduction in sclerotic lesions.^9,10^ To date, four genes have been reported as causal genes of DOS, including solute carrier family 29 member 3 (*SLC29A3*),^11^ tumor necrosis factor receptor superfamily member 11a (*TNFRSF11A*),^8^ T cell immune regulator 1 (*TCIRG1*)^12^ and colony stimulating factor 1 receptor (*CSF1R*).^13^ Despite involving different pathways, these genes are all associated with impaired osteoclast differentiation and/or function, leading to reduced bone resorption and disrupted bone remodeling.

Erythropoietin-producing hepatocellular (EPH) receptors are the largest family of receptor tyrosine kinases activated by ligands called EPH receptor interacting proteins (ephrins).^14–16^ In recent years, accumulating evidence supports their roles in bone homeostasis, with most studies focusing on EPHB/ephrin-B bidirectional signaling.^17–20^ EPHA2, a protein in the EPHA receptors family encoded by the *EPHA2* gene, has become a hot topic of research due to its role in tumors^21,22^ and cataracts (OMIM 176946).^23,24^ However, less is known about the role of EPHA2 in bone homeostasis. More recently, a proteome-wide Mendelian randomization study nominated ephrin-A1–EPHA2 signalling as a potential fracture-preventive pathway and reported that EPHA2 expression is among the highest in both cortical and trabecular bone; however, the causal role of EPHA2 in skeletal homeostasis and the underlying cellular mechanisms remain to be established.^25^ Irie et al. showed at the cellular level that EPHA2/ephrin-A2 bidirectional signaling enhances osteoclastogenesis and EPHA2 signaling inhibits osteoblast differentiation.^26^ Liu et al. also found that knockdown of EPHA2 or ephrin-A2 in rat bone marrow-derived macrophages (BMMs) significantly impaired osteoclastogenesis.^27^ Ren et al. found that adipogenic capacity was significantly elevated, and mineralization capacity was markedly reduced in rat bone marrow mesenchymal stem cells (BMSCs) after overexpression of EPHA2 alone.^28^ All of the above studies suggest that EPHA2 may be an important regulator in bone homeostasis. However, there have been no reports associating *EPHA2* with genetic skeletal disorders and the underlying molecular mechanisms remain to be elucidated.

In the present study, we report a Chinese patient with DOS phenotype characterized by extremely high bone mineral density (BMD), focal sclerosis, sclerotic skull, platyspondyly and sandwich vertebrae, resulting from compound heterozygous mutations in *EPHA2*. We elucidated the molecular mechanism of its pathogenesis by in vitro cellular assays, genome-wide association studies (GWAS) analysis, induced pluripotent stem cells (iPSCs) models and *Epha2* knockout mice models. The roles of *Epha2* in osteoporosis and bone fracture models were further elaborated, suggesting that EPHA2 is a potential therapeutic target for osteoporosis, especially for severe osteoporosis combined with fragility fractures.

## Results

### Compound heterozygous mutations in *EPHA2* causes a new type of DOS

**Fig. 1:**
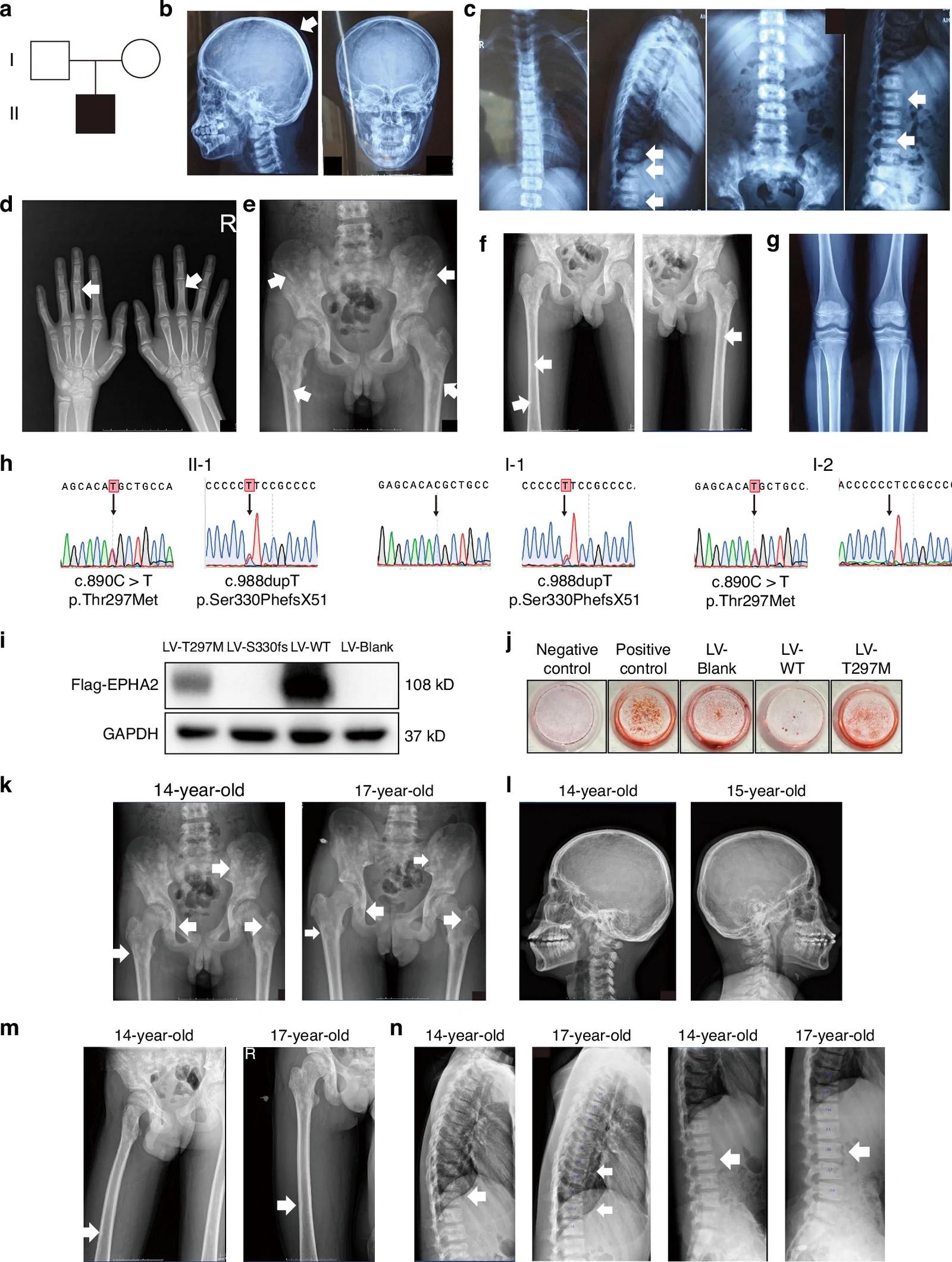
Radiological findings and functional validation of mutations. **a** Pedigree of the family. **b** Anteroposterior and lateral skull X-ray examination of the patient at 12-year-old: diffuse osteosclerosis, which is prominent in the skull base. **c** Anteroposterior and lateral X-ray examination of the thoracic and lumbar spine of the patient at 12-year-old: platyspondyly with focal or patchy sclerosis in the vertebral endplates. **d** Bilateral hand X-ray examination of the patient at 14-year-old: punctate sclerosis of phalanges without metaphyseal widening. **e** Pelvic X-ray examination of the patient at 14-year-old: focal sclerosis of iliac bone and proximal femur. **f** Femoral X-ray examination of the patient at 14-year-old: focal sclerosis of the proximal femur and the distal cortical bone. **g** Knee joint X-ray examination of the patient at 12-year-old: absence of metaphyseal widening and sclerosis. **h** The Sanger sequencing results of *EPHA2* of the family. **i** Western blot results following plasmids transfection of HEK293T cells. **j** Following lentiviral transduction of human BMSCs with the plasmids and subsequent osteogenic induction, the results of the ARS staining for osteoblasts. **k** Pelvic X-ray examination of the patient at 14-year-old and 17-year-old. **l** Skull X-ray examination of the patient at 14-year-old and 15-year-old. **m** Femoral X-ray examination of the patient at 14-year-old and 17-year-old. **n** Thoracic spine and lumbar spine X-ray examination of the patient at 14-year-old and 17-year-old. The areas exhibiting significant alterations are indicated by the arrows

We found a patient with extremely high BMD born to non-consanguineous unaffected parents. This patient (Ⅱ-1 in Fig. 1a) is a 12-year-old male who presented to our department in October 2019 due to relatively lower height compared with age-matched peers during routine growth monitoring (145 cm, 34 kg; both corresponding to the 10^th^–25^th^ percentile^29^). The patient had an unremarkable prenatal and postnatal course. Parental history suggested an unsteady gait during early childhood (around 2 years of age). At presentation, the patient exhibited normal mobility and daily activity, which remained stable throughout the subsequent follow-up period. The patient presented with no history of fractures, and exhibited normal hearing. Refractive examination indicates astigmatism: right eye (OD): plano sphere, −2.75 D cylinder at axis 180°; left eye (OS): plano sphere, −2.75 D cylinder at axis 170° (Table S1). Dual-energy X-ray absorptiometry (DXA) suggested the significant increase in BMD, with the lumbar spine BMD of 1.114 g/cm^2^ (*Z*-score = 3.7^30^), the femoral neck BMD of 0.860 g/cm^2^ (*Z*-score = 1.1), and the total hip BMD of 0.904 g/cm^2^ (*Z*-score = 1.1, Table 1). Radiological examination revealed craniofacial bones osteosclerosis (Fig. 1b), platyspondyly with focal or patchy sclerosis in the vertebral endplates (Fig. 1c), striated sclerosis of phalanges (Fig. 1d), striated and punctate sclerosis of iliac bone (Fig. 1e) and focal sclerosis of the proximal femur and the distal cortical bone (Fig. 1e, f). However, metaphyseal sclerosis and widening were not observed (Fig. 1d, g). Biochemical indicators and bone turnover biomarkers (BTMs) are shown in Table 2. The circulating levels of carboxy-terminal cross-linked telopeptide of type I collagen (CTX) and osteocalcin (OC) were 2 950 ng/L and 87.82 ng/mL, respectively, which were within the age- and sex-matched reference ranges, with CTX close to the upper limit.^31^ Focal sclerosis, sclerotic skull, platyspondyly and sandwich vertebrae in this patient strongly support a diagnosis of DOS.

**Table 1:** Bone mineral density at visit and follow-up

Family member	Date	Age/year	Height/cm	Weight/kg	L1-4/(g/cm^2^, *Z*-score or *T*-score)	FN/g/cm^2^, *Z*-score or *T*-score)	TH/g/cm^2^, *Z*-score or *T*-score)
Patient (Ⅱ-1)	October 2019	12	145.3	34	1.114 (3.7)	0.860 (1.1)	0.904 (1.1)
	July 2021	14	156.2	38	1.197 (3.7)	0.904 (1.1)	0.985 (1.2)
	August 2022	15	164.2	44	1.307 (4.7)	0.894 (1.0)	0.968 (1.1)
	July 2023	16	169.2	50	1.419 (5.2)	0.978 (1.2)	1.059 (1.4)
	August 2024	17	171.2	52	1.517 (6.1)	0.999 (1.4)	1.083 (1.6)
Patient’s father (Ⅰ-1)	October 2019	37	178	78	0.885 (−1.8)	0.759 (−0.9)	0.812 (−1.3)
Patient’s mother (Ⅰ-2)	October 2019	38	160	54	1.114 (0.8)	0.744 (−0.7)	0.857 (−0.5)

*L1-4* Lumbar spine 1-4, *FN* Femoral neck, *TH* Total hip

**Table 2:** Biochemical indicators and bone turnover biomarkers at visit and follow-up

Date	Ca/(mmol/L)	P/(mmol/L)	ALP/(U/L)	25(OH)D/(ng/mL)	PTH/(pg/mL)	CTX/(ng/L)	OC/(ng/mL)	P1NP/(ng/mL)
October 2019	2.36	1.57	225	16.13	35.87	2950	87.82	-
July 2021	2.33	1.65	383	14.82	44.63	-	-	-
August 2022	2.37	1.70	286	16.66	26.17	4074	119.40	971.60
July 2023	2.27	1.6	242	19.11	48.74	3765	112.20	791.70
August 2024	2.26	1.55	200	14.24	55.59	3270	65.48	563.50
Reference range* (boys, > 12 years): CTX 663.6–2 996.0 ng/L; OC 19.0–143.6 ng/mL; P1NP 117.6–1 532.1 ng/mL.								

^*^Reference ranges are age- and sex-matched values derived from Chinese pediatric populations using the same analytical methods^31^

*ALP* alkaline phosphatase, *25(OH)D* 25-hydroxy vitamin D, *PTH* parathyroid hormone, *CTX* carboxy-terminal cross-linked telopeptide of type 1 collage, *OC* osteocalcin, *P1NP* procollagen type 1 N-terminal propeptide

Both parents of the patient denied any history of fractures with normal height and BMD, as detailed in Table 1. However, the patient’s mother (Ⅰ-2) has experienced progressive visual impairment since the age of 42, specifically manifesting as blurred vision and eye fatigue after 30 min of sustained near vision use, accompanied by difficulty seeing in dim light. Previous corrective lens prescriptions have not provided satisfactory refractive correction. Refractive examination revealed myopia, astigmatism, and presbyopia in both eyes, and the corrected visual acuity in both eyes is reduced (Table S1). Slit-lamp microscopy revealed a wedge-shaped opacity in the left eye’s lens cortex (Fig. S1), leading to a clinical diagnosis of cortical cataract. The patient’s father has normal vision (Table S1), and slit-lamp examination revealed no abnormalities (Fig. S1).

To identify the causative gene, we performed trio whole-exome sequencing (WES) after excluding known genes associated with sclerosing bone dysplasias. After bioinformatic analysis (Table S2) and validation by Sanger sequencing, we identified compound heterozygous variants in the *EPHA2* gene (NM_004431.5) in the patient: c.890 C > T (p.Thr297Met) and c.988dupT (p.Ser330PhefsX51), both previously reported and are listed in the ClinVar database. Furthermore, his father had a heterozygous mutation in *EPHA2* S330fs and his mother had a heterozygous mutation in *EPHA2* T297M, as shown in Fig. 1h. The frameshift variant c.988dupT is predicted to generate a premature termination codon and thereby trigger nonsense-mediated mRNA decay, leading to absence of functional EPHA2 protein. In line with this prediction, *EPHA2* S330fs failed to produce detectable protein upon overexpression in HEK293T cells, confirming a loss-of-function effect (Fig. 1i). However, the effect of T297M on protein function is unknown. As illustrated in Fig. 1i, T297M also significantly reduces the protein expression of EPHA2. Furthermore, we overexpressed *EPHA2* T297M in human BMSCs, induced into osteoblasts and verified the function of this mutation by Alizarin Red S (ARS) staining (Fig. 1j). Prior research has demonstrated that EPHA2 inhibits osteogenesis.^26^ However, the T297M mutation abrogates this inhibitory effect, suggesting that T297M is a loss-of-function mutation.

Subsequently, we followed up this patient annually for five years to assess growth and longitudinal changes in sclerotic lesions as shown in Tables 1–2 and Fig. 1k–n. With pubertal development, the patient’s BMD increased annually, especially in the lumbar spine. As shown in Fig. 1k, the sclerotic lesions in the pelvis and proximal femur have decreased, with some areas showing complete resolution. The degree of cranial sclerosis was significantly reduced, with the skull base being particularly affected (Fig. 1l). The striated sclerosis of the distal femoral cortex was also attenuated (Fig. 1m). Sclerosis of the vertebral endplates was attenuated, while vertebral bodies exhibited increased flattening and more pronounced marginal osteophyte (Fig. 1n). Overall, in the past five years, the patient’s multiple sclerotic lesions in the pelvis, femur, and vertebral bodies have decreased, especially in the skull, which is consistent with the regression of DOS sclerotic lesions reported in previous literature.^9,10^

### Inhibited osteoclastogenesis and enhanced osteoblastogenesis in the patient

**Fig. 2:**
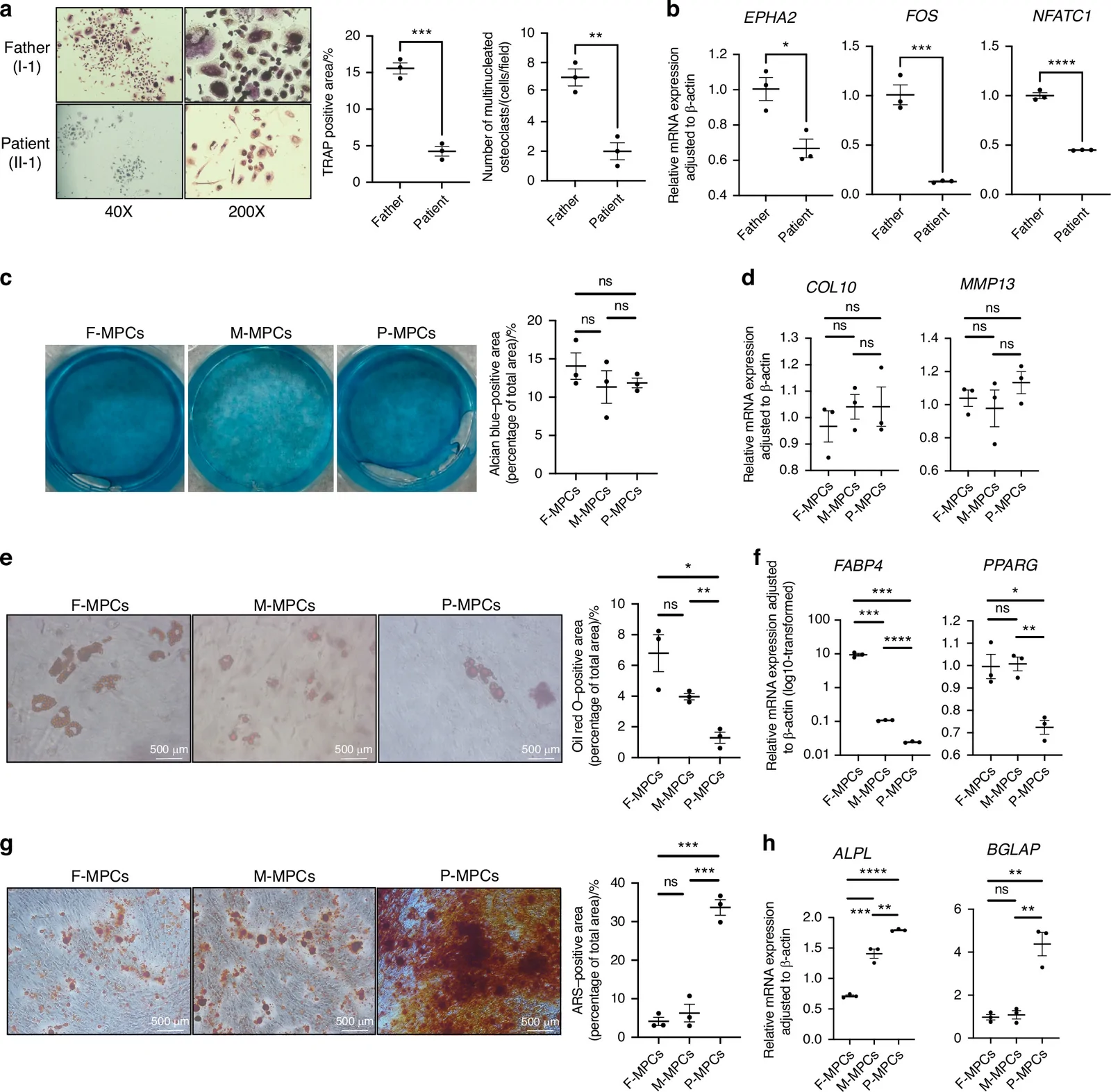
Inhibited osteoclastogenesis and enhanced osteoblastogenesis in the patient. **a** Osteoclast differentiation of peripheral blood mononuclear cells between patients and his father, and three wells per donor were analyzed as technical replicates. **b** qRT-PCR results of key osteoclastogenesis genes (repeated in three independent experiments). **c** Alcian blue staining after MPCs chondrogenic differentiation. **d** qRT-PCR results of key chondrogenic differentiation genes. **e** Oil red O staining after MPCs adipogenic differentiation. **f** qRT-PCR results of key adipogenic differentiation genes. **g** ARS staining after MPCs osteogenic differentiation. **h** qRT-PCR results of key osteogenic differentiation genes. iPSCs- and MPCs-based assays were quantified using three independent iPSC clones for statistical analysis

To investigate the formation of osteoblasts and osteoclasts in the patient, we employed the following two methods. Initially, peripheral blood mononuclear cells (PBMCs) from the patient and his father were differentiated into osteoclasts and assessed by tartrate-resistant acid phosphatase (TRAP) staining (Fig. 2a). The patient had significantly fewer osteoclasts than his father, suggesting impaired osteoclast formation. qRT-PCR further showed significantly reduced expression of *EPHA2*, *FOS*, and *NFATC1* in patient-derived osteoclasts (Fig. 2b). Subsequently, we generated iPSCs models from the patient and his parents and differentiated them into mesenchymal progenitor cells (MPCs). Patient-, father-, and mother-derived iPSCs (P-/F-/M-iPSCs) expressed high levels of pluripotency markers (*POU5F1*, *NANOG*, and *SOX2*), displayed normal morphology and karyotype (Fig. S2a–c), and carried the correct *EPHA2* gene mutation (Fig. S2d). As illustrated in Fig. 2c–h, F-, M-, and P-MPCs are all capable of differentiating into adipogenic, osteogenic, and chondrogenic lineages. Upon osteogenic induction, P-MPCs displayed significantly enhanced mineralization compared with parental MPCs (Fig. 2g), consistent with increased expression of osteogenic markers *ALPL* and *BGLAP* by qRT-PCR (Fig. 2h). In contrast, Oil Red O staining and adipogenic marker expression indicated reduced adipogenic differentiation of P-MPCs (Fig. 2e, f), whereas chondrogenic induction showed no obvious difference among groups (Fig. 2c, d).

### Genetic association of *EPHA2* with BMD

To further investigate the association of the *EPHA2* gene with BMD in humans, we extracted the genetic association estimates of this gene region (approximately ±250 kb of the gene region, based on hg19 coordinates, chr1: 16200832-16732582) for DXA derived-BMD at 11 skeletal sites (i.e., arm, total femur, femoral neck, head, legs, lumbar spine, pelvis, ribs, spine, trunk, and total body BMD) from our previous GWAS results^32^ (Table S3). We then meta-analyzed these genetic association estimates by cross-phenotype association analysis (CPASSOC). Among 2 198 genetic variants within the *EPHA2* gene region, 18 single nucleotide polymorphisms (SNP) were identified to be associated with BMD at a Bonferroni-corrected significant level (*P*-value < 1.68 × 10^−^^5^), of which, SNP rs778478101 represented the top signal (*P*-valueShet = 9.73 × 10^−11^) (Fig. S3).

### *Epha2*^−^^*/*^^−^ mice exhibit an increased bone mass phenotype and abnormal bone metabolism

**Fig. 3:**
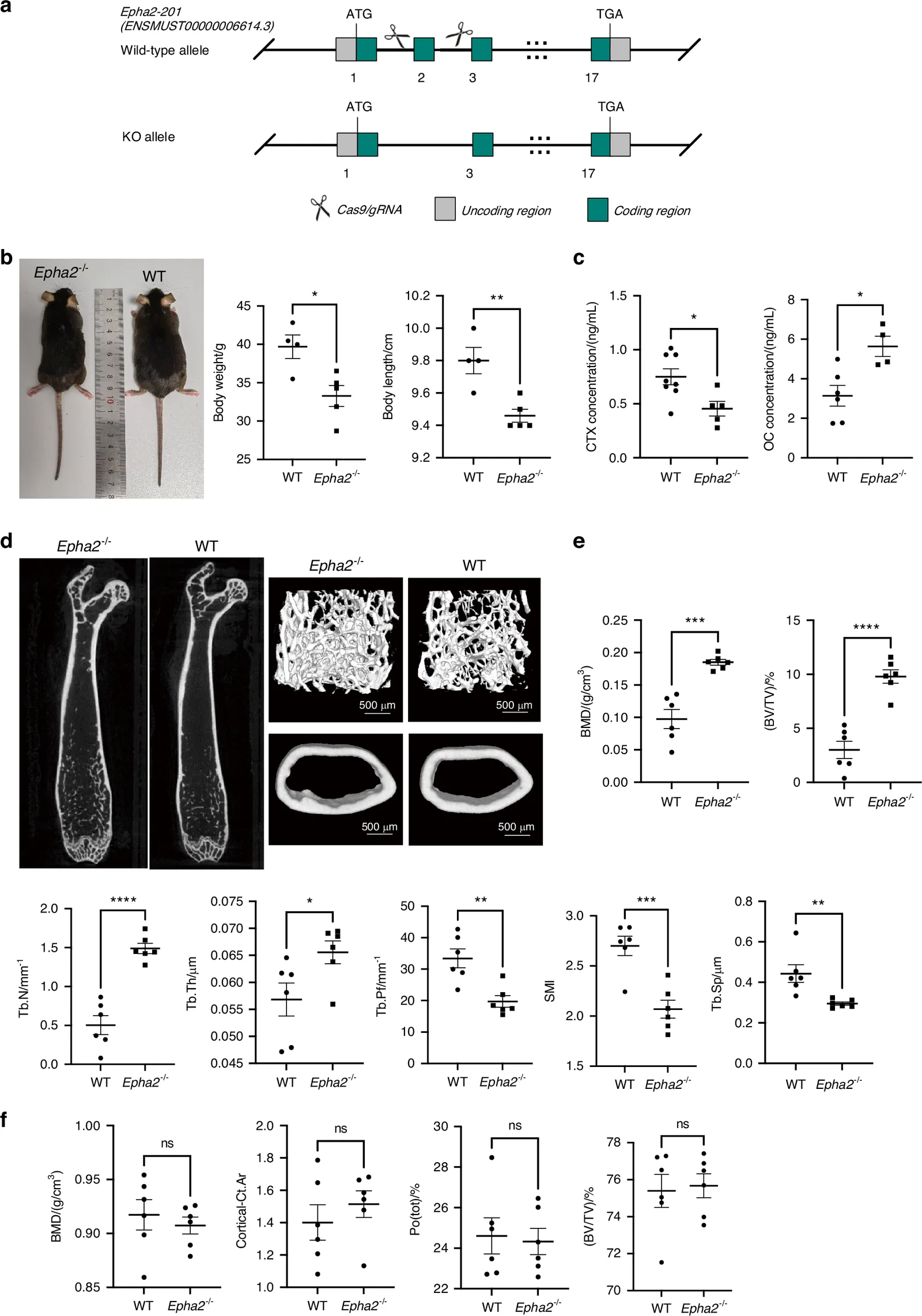
*Epha2*^*−/−*^ mice exhibit an increased bone mass phenotype and abnormal bone metabolism. **a** *Epha2*^*−/−*^ mice model construction strategies. **b** Comparison of body weight and body length between the 12-month-old male *Epha2*^*−/−*^ mice (*n* = 5) and WT mice (*n* = 4). **c** Comparison of the BTMs between 12-month-old male *Epha2*^*−/−*^ mice (CTX, *n* = 5; OC, *n* = 4) and WT mice (CTX, *n* = 8; OC, *n* = 6) measured by ELISA. **d** Micro-CT 2D images and 3D reconstructions of 12-month-old male *Epha2*^*−/−*^ mice and WT mice. **e** Results of trabecular bone parameter analysis (*n* = 6). **f** Results of cortical bone parameter analysis (*n* = 6)

To further investigate the pathogenesis of *EPHA2* leading to DOS, we constructed *Epha2*^−/−^ mice using CRISPR/Cas9 (Fig. 3a and Fig S4a, b). At 12 months, male *Epha2*^*−/−*^ mice showed reduced body length and weight compared with WT littermates (Fig. 3b). In body composition analysis at 10 months, fat mass was not significantly different between genotypes (Fig. S4c). Subsequently, we assessed BMD across multiple ages (3, 6, 12, and 14 months) (Fig. S5) and observed a clear skeletal phenotype in 12-month-old males. In these mice, serum BTMs measured by ELISA showed decreased CTX (bone resorption) and increased osteocalcin (OC; bone formation) compared with WT (Fig. 3c), indicating reduced resorption and enhanced formation. Micro-CT analysis revealed an increased bone mass phenotype in 12-month-old male *Epha2*^*−/−*^ mice (Fig. 3d, e), with increased trabecular BMD, bone volume/tissue volume (BV/TV), trabecular number (Tb.N) and trabecular thickness (Tb.Th), and decreased trabecular pattern factor (Tb.Pf), structure model index (SMI), and trabecular separation (Tb.Sp). However, cortical parameters were unchanged (Fig. 3f).

### Imbalance between osteogenic and adipogenic differentiation of MSCs in *Epha2*^*−/−*^ mice

**Fig. 4:**
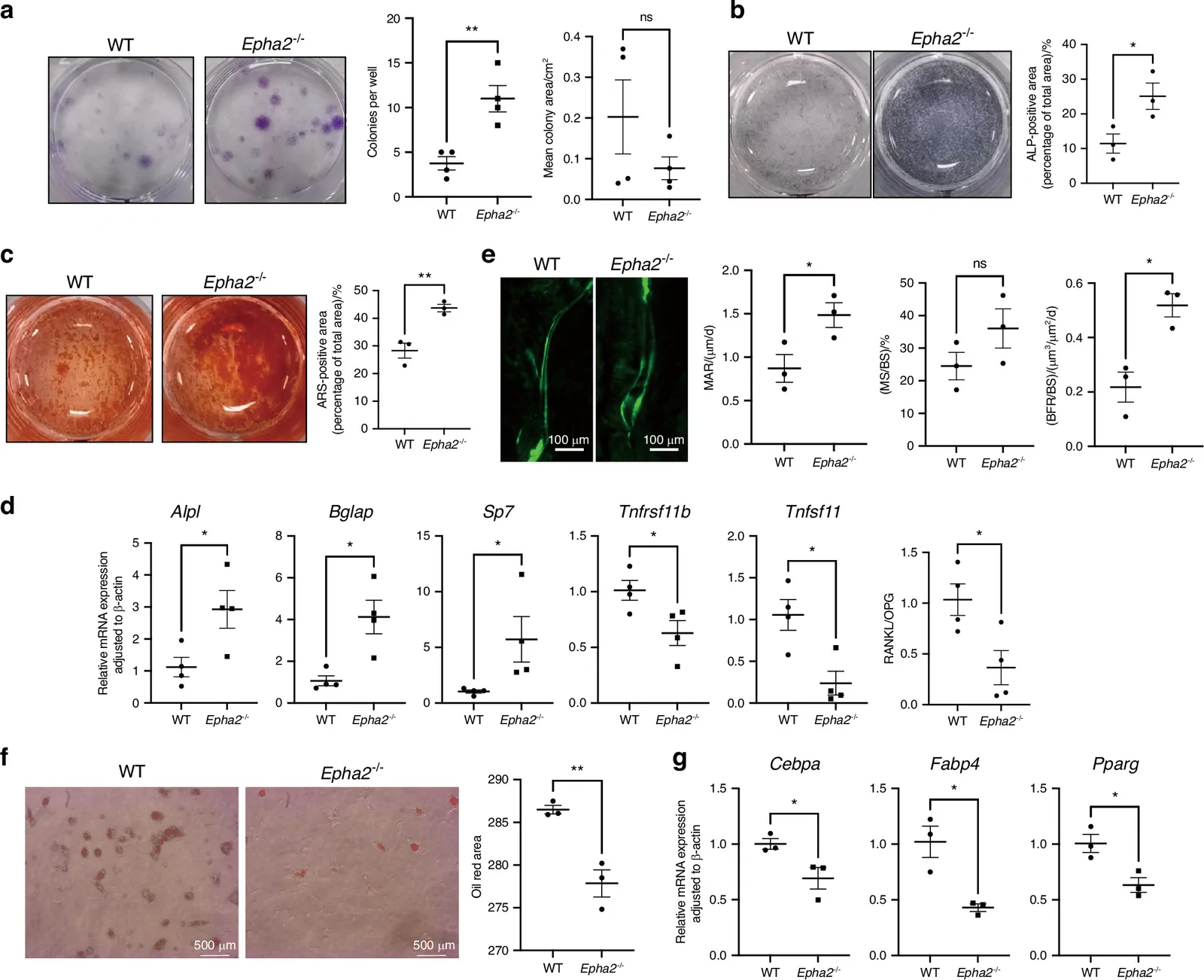
Imbalance between adipogenic and osteogenic differentiation of MSCs in *Epha2*^*−/−*^ mice. **a** CFU-F assay for BMSCs derived from 14-week-old *Epha2*^*−/−*^ mice and WT mice (*n* = 4). **b** ALP staining of osteoblasts from 14-week-old *Epha2*^*−/−*^ mice and WT mice (*n* = 3). **c** ARS staining of osteoblasts from 14-week-old *Epha2*^*−/−*^ mice and WT mice (*n* = 3). **d** qRT-PCR results of key genes involved in osteogenic differentiation of 14-week-old *Epha2*^*−/−*^ mice and WT mice (*n* = 4). **e** Calcein double labeling assay of 10-month-old *Epha2*^*−/−*^ mice and WT mice (*n* = 3). **f** Oil red O staining for BMSCs adipogenic differentiation of 14-week-old *Epha2*^*−/−*^ mice and WT mice (*n* = 3). **g** qRT-PCR results of key genes involved in adipogenic differentiation of 14-week-old *Epha2*^*−/−*^ mice and WT mice (*n* = 3)

To investigate the molecular mechanism of increased BMD in *Epha2*^*−/−*^ mice, we first extracted BMSCs from 14-week mice and performed colony-forming unit fibroblasts (CFU-F) assay. *Epha2*^*−/−*^ cultures formed more CFU-F colonies than WT, supporting an increased colony-forming frequency of stromal progenitors, while mean colony area was unchanged (Fig. 4a). Then, under osteogenic conditions, alkaline phosphatase (ALP) and ARS staining revealed that osteoblasts of *Epha2*^*−/−*^ mice exhibited enhanced differentiation than of WT (Fig. 4b, c), accompanied by increased expression of *Alpl*, *Bglap* and *Sp7* (Fig. 4d). However, the expression levels of *Tnfrsf11b* (encoding OPG) and *Tnfsf11* (encoding RANKL) were decreased in *Epha2*^*−/−*^ osteoblasts, indicating an altered RANKL/OPG axis (Fig. 4d). Osteoblast activity was further verified by calcein double labeling assay. As shown in Fig. 4e, 10-month-old *Epha2*^*−/−*^ mice had an enhanced mineral apposition rate (MAR) and bone formation rate/BS (BFR/BS) compared with WT, without a significant change in mineralizing surface (MS/BS). These results indicated enhanced osteoblast differentiation and osteoblast activity in *Epha2*^*−/−*^ mice.

Since BMSCs differentiate into osteoblasts and adipocytes in the competing and reciprocal pathway, and their normal function is important for the balance of bone metabolism.^33^ We subsequently validated BMSCs adipogenic capacity in *Epha2*^*−/−*^ mice. BMSCs from 14-week mice were extracted and cultured in adipogenesis induction medium. Oil red O staining was performed 7 days after induction (Fig. 4f). The formation of lipid droplets in *Epha2*^*−/−*^ mice was significantly weaker than in WT. Meanwhile, the mRNA levels of CCAAT/enhancer-binding protein alpha (*Cebpa*), fatty acid binding protein 4 (*Fabp4*) and peroxisome proliferator-activated receptor gamma (*Pparg*), which were the important markers of adipocyte differentiation, were also inhibited (Fig. 4g).

### Impaired osteoclast differentiation resulting from the downregulation of the p38 MAPK pathway in *Epha2*^*−/−*^ mice

**Fig. 5:**
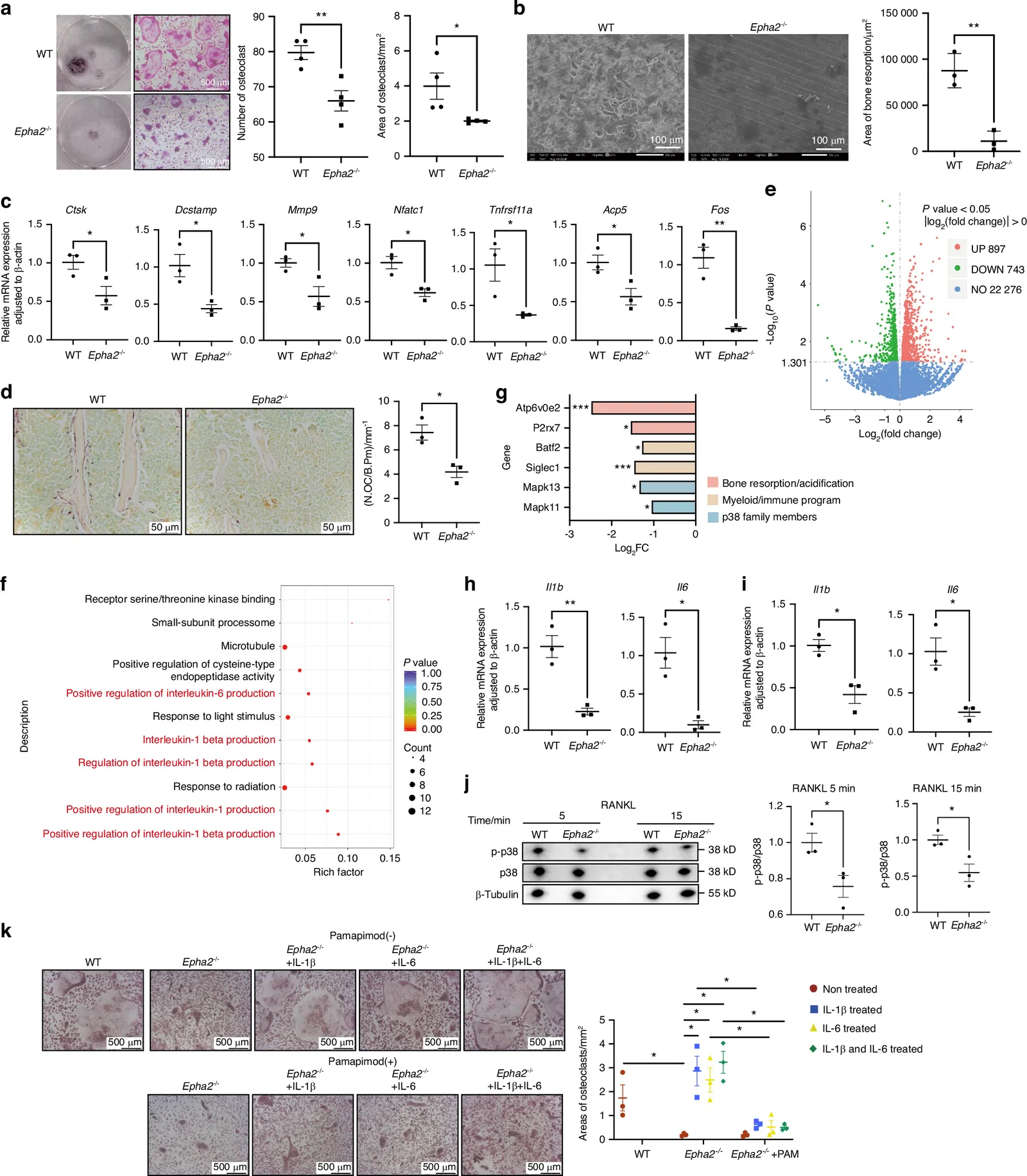
Impaired osteoclast differentiation resulting from the downregulation of the p38 MAPK pathway in *Epha2*^*−/−*^ mice. **a** TRAP staining of osteoclasts from 14-week-old *Epha2*^*−/−*^ mice and WT mice (*n* = 4). **b** Pit formation assay of osteoclasts from 14-week-old *Epha2*^*−/−*^ mice and WT mice (*n* = 3). **c** qRT-PCR results of key genes involved in osteoclast differentiation of 14-week-old *Epha2*^*−/−*^ mice and WT mice (*n* = 3). **d** TRAP staining of femur sections of 12-month-old *Epha2*^*−/−*^ mice and WT mice (*n* = 3). **e** Volcano plot of differentially expressed genes between WT and *Epha2*^*−/−*^ osteoclasts. **f** Bubble chart of GO enrichment analysis between WT and *Epha2*^*−/−*^ osteoclasts. **g** Bar plot of representative differentially expressed genes. **h** qRT-PCR results of osteoblasts of 14-week-old *Epha2*^*−/−*^ mice and WT mice (*n* = 3). **i** qRT-PCR results of bone tissue of 23-week-old *Epha2*^*−/−*^ mice and WT mice (*n* = 3). **j** Western blot results of BMMs of WT and *Epha2*^*−/−*^ mice at 5 and 15 min following RANKL stimulation (*n* = 3). **k** The effects of IL-1β, IL-6, and pamapimod on *Epha2*^*−/−*^ osteoclast differentiation (*n* = 3). For panel (**g**), significance was determined by FDR-adjusted *P* values (*FDR < 0.05, **FDR < 0.01, ***FDR < 0.001)

Subsequently, we extracted bone marrow cells from 14w mice and induced them into osteoclasts. TRAP staining suggested that the number and area of osteoclasts in *Epha2*^*−/−*^ mice were significantly lower than in WT (Fig. 5a). Consistently, *Epha2*^*−/−*^ mice osteoclasts resorbed a smaller area of bone surface compared with WT (Fig. 5b). Results of qRT-PCR suggested down-regulation of critical genes for osteoclast differentiation, namely *Ctsk*, *Dcstamp*, *Mmp9, Nfatc1*, *Tnfrsf11a*, *Acp5* and *Fos* (Fig. 5c). To quantify osteoclasts, we used the TRAP staining again on femur sections of 12-month-old mice. As shown in Fig. 5d, the number of osteoclasts per bone surface was lower in *Epha2*^*−/−*^ mice compared with WT. Masson staining of undecalcified femoral tissue sections also indicated a reduction in the number of osteoclasts per bone surface and an increase in the number of osteoblasts per bone surface in *Epha2*^*−/−*^ mice (Fig. S6a). These results indicated impaired osteoclast differentiation capacity and diminished bone resorption in *Epha2*^*−/−*^ mice.

To link the osteoblast and osteoclast phenotypes, we performed osteoblast lineage cells (OLC)-BMM co-culture assay and quantified the numbers and areas of osteoclasts. Compared with WT, co-cultures involving *Epha2*^*−/−*^ BMSC or OLC generated fewer osteoclasts (Fig. S6b). These results support a coupled role of EPHA2 in bone remodeling, with EPHA2 deficiency affecting both the osteoclastogenesis and OLC support.

To detect the underlying mechanisms of the impaired osteoclastogenesis, the whole transcriptome of WT and *Epha2*^*−/−*^ osteoclasts was analyzed. Among the significantly and differentially expressed transcripts, 743 genes were down regulated (*P* < 0.05), and 897 genes were upregulated in *Epha2*^*−/−*^ osteoclasts compared with WT (Fig. 5e). Interestingly, Gene Ontology (GO) enrichment analysis revealed that regulations of biological process of interleukin-1 beta (IL-1β) and interleukin-6 (IL-6) were strongly correlated with differentially expressed mRNAs between WT and *Epha2*^*−/−*^ osteoclasts (Fig. 5f). Moreover, genes related to the bone resorption/acidification, myeloid/immune program, and p38 family members were significantly downregulated in the *Epha2*^*−/−*^ group (Fig. 5g). Therefore, we assessed the expression levels of IL-1β and IL-6 in osteoblast and bone tissue using qRT-PCR. As shown in Fig. 5h, i, the expression levels of both cytokines were significantly reduced in osteoblasts and bone tissue, respectively. IL-1β and IL-6 can stimulate osteoblasts/bone stromal cells to secrete RANKL, thereby upregulating the nuclear factor-kappa B (NF-κB) and mitogen-activated protein kinase (MAPK) family to promote osteoclast differentiation and bone resorbing activity of mature osteoclasts.^34^ Given the differential expression of p38 family members, we further validated the alterations in p38-MAPK pathway via western blot. As shown in Fig. 5j, following RANKL induction, the p38-MAPK pathway is significantly suppressed in *Epha2*^*−/−*^ mice at both 5 and 15 min. To further validate the role of p38-MAPK pathway in *Epha2*^*−/−*^ osteoclast differentiation, we rescued the osteoclast phenotype by supplementing IL-1β and/or IL-6 during osteoclast differentiation. As shown in Fig. 5k, the addition of IL-1β and/or IL-6 to *Epha2*^*−/−*^ osteoclast differentiation reversed the impaired osteoclastogenesis phenotype. However, this rescue effect was abolished upon the addition of pamapimod (PAM, the p38-MAPK inhibitor).

### Accelerated bone fracture healing in *Epha2*^*−/−*^ mice

Fracture is a common clinical manifestation of sclerosing bone dysplasias. However, our patient had no history of fracture. Therefore, to further investigate the role of the *EPHA2* gene in fracture, we constructed femoral fracture models in *Epha2*^*−/−*^ mice and WT mice. As shown in Fig. S7a, the healing callus density of *Epha2*^*−/−*^ mice at 14, 21, and 28 days after the fracture was higher than that of WT mice. Micro-CT analysis of the callus at 28 days after the fracture also indicated that fracture healing was superior in *Epha2*^*−/−*^ mice compared to WT mice (Fig. S7b, c).

### *EPHA2* is a potential therapeutic target for osteoporosis

**Fig. 6:**
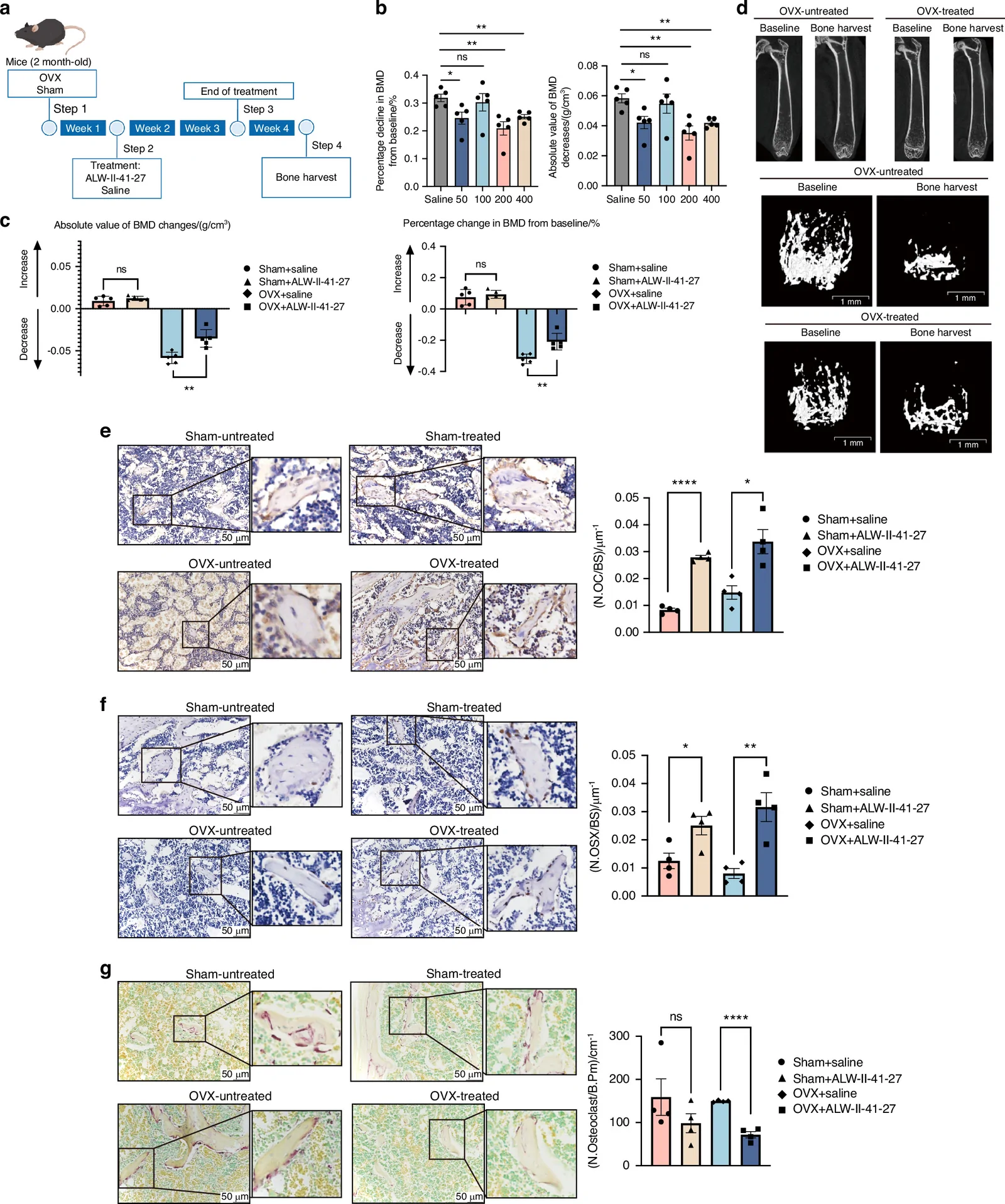
EPHA2 inhibition serves as a potential therapeutic target against osteoporosis. **a** Experimental flowchart for OVX mice model. **b** Absolute and relative decreases in BMD following treatment with varying concentrations of ALW-II-41-27 (50, 100, 200, and 400 μg/kg) in OVX mice, with saline as the control (*n* = 5). **c** Absolute and relative changes in BMD among the Sham-untreated (saline), Sham-treated (ALW-II-41-27; 200 μg/kg), OVX-untreated (saline), and OVX-treated groups (ALW-II-41-27; 200 μg/kg) (*n* = 5). **d** Micro-CT reconstruction images of OVX-untreated and OVX-treated groups. **e** Immunohistochemical analysis of osteocalcin (*n* = 4). **f** Immunohistochemical analysis of osterix (*n* = 4). **g** TRAP staining of femur sections (*n* = 4)

Based on the patient phenotypes, GWAS analysis, iPSC models, and *Epha2*^*−/−*^ mice phenotypes, we propose that *EPHA2* may be a potential therapeutic target for osteoporosis. Therefore, we established the ovariectomy (OVX) mice models in 8-week-old female mice and treated animals with the EPHA2 inhibitor ALW-II-41-27 (Fig. 6a). A dose-ranging study (50, 100, 200, and 400 μg/kg) identified 200 μg/kg as the dose associated with the least OVX-induced BMD loss (Fig. 6b). Subsequently, we compared the changes in BMD among the Sham-untreated, Sham-treated, OVX-untreated, and OVX-treated groups using 200 μg/kg. As illustrated in Fig. 6c, BMD increased from baseline in the sham group mice, with no significant difference between Sham-untreated and Sham-treated groups. Conversely, BMD decreased from baseline in the OVX group mice, although the OVX-treated group exhibited significantly less bone loss compared to the OVX-untreated group (Fig. 6c, d). Immunohistochemical analysis of osteogenic differentiation proteins, OC and osterix (OSX, encoded by *Sp7*), revealed that ALW-II-41-27 treatment significantly increased the number of OC-positive cells (Fig. 6e) and OSX-positive cells (Fig. 6f) per bone surface. TRAP staining indicated a significant reduction in the number of osteoclasts per bone surface following ALW-II-41-27 treatment in the OVX group mice (Fig. 6g). Given that ALW-II-41-27 promotes osteogenic differentiation and inhibits osteoclast differentiation, thereby reducing bone loss in OVX mice, supporting *EPHA2* as a potential therapeutic target for osteoporosis.

## Discussion

To date, emerging studies have indicated that *EPHA2* played a crucial role in bone metabolism. However, there have been no reports associating *EPHA2* with genetic skeletal disorders. Here, we reported a Chinese patient with DOS caused by the *EPHA2* gene biallelic loss-of-function mutations, characterized by markedly elevated lumbar spine BMD (*Z*-score = 6.1), craniofacial bones osteosclerosis, platyspondyly and focal sclerosis throughout the body. Then, we found that *EPHA2* loss-of-function mutations increase bone mass by enhancing MSC osteogenic differentiation and downregulating the p38-MAPK pathway, leading to reduced osteoclast differentiation. Based on these findings, pharmacological inhibition of EPHA2 mitigated OVX-induced bone loss, supporting *EPHA2* as a potential therapeutic target for osteoporosis.

This Chinese patient with *EPHA2* gene compound heterozygous mutations presented key characteristics of DOS, including extremely elevated lumbar spine BMD, craniofacial bones osteosclerosis, platyspondyly and focal sclerosis throughout the body, particularly in the pelvis and at the endplates of the vertebral bodies. The regression of the patient’s sclerotic lesions during follow-up further validates our diagnosis. However, *EPHA2* gene mutations exhibit distinct characteristics compared to DOS caused by other genes. Firstly, our patient has no history of fractures, and *Epha2*^*−/−*^ mice exhibit accelerated fracture healing, suggesting that fracture susceptibility in *EPHA2*-associated DOS may differ from other forms of DOS. Secondly, unlike *SLC29A3*-associated DOS, metaphyseal widening or a radiolucent zone above it was not observed in this patient. Thirdly, at the most recent follow-up, the patient’s height had reached a normal adult height (171.2 cm). Similar cases with normal adult height have been reported by Guo et al.^13^ Fourthly, unlike previously reported DOS genes that predominantly impair osteoclast differentiation and/or function, *EPHA2* acts as a bidirectional regulator of bone remodeling and also affects fracture healing, suggesting a distinct mechanism beyond an osteoclast-only defect. Notably, the skeletal phenotype appears to preferentially involve trabecular bone. The patient exhibited a significant increase in lumbar spine BMD (*Z*-score = 6.1; primarily trabecular bone) while femoral neck BMD remained within normal level (*Z*-score = 1.4; primarily cortical bone). Consistently, 12-month-old male *Epha2*^*−/−*^ mice exhibited significantly increased trabecular BMD and microarchitecture compared to WT mice, while cortical bone showed no significant differences. However, given that this paper only presents one case of *EPHA2*-associated DOS, the phenotype spectrum requires further refinement with the inclusion of more patient reports.

*EPHA2* is also associated with multiple types of cataracts (OMIM 116600), which are inherited in an autosomal dominant manner, including congenital cataract and age-related cataracts.^35^ The majority of cataract-related *EPHA2* mutations are missense mutations, which are frequently within/close to the intracellular Sterile alpha motif (SAM) domain.^36^ In our family, the mother was diagnosed with age-related cataracts, carried the T297M mutation, while the father, with normal vision, carried the S330fs mutation. The absence of cataract in the father may be due to incomplete penetrance. Jun et al. also reported a *EPHA2* Arg721Gln mutation carrier but remained free of cataract at age 63.^37^ Our patient, while not presenting with cataracts, does exhibit severe astigmatism (-2.75D). This may be due to *EPHA2* mutations leading to disorganization of lens fiber cell architecture, which in turn causes asymmetric curvature of the lens surface and aberrant light refraction.

**Fig. 7:**
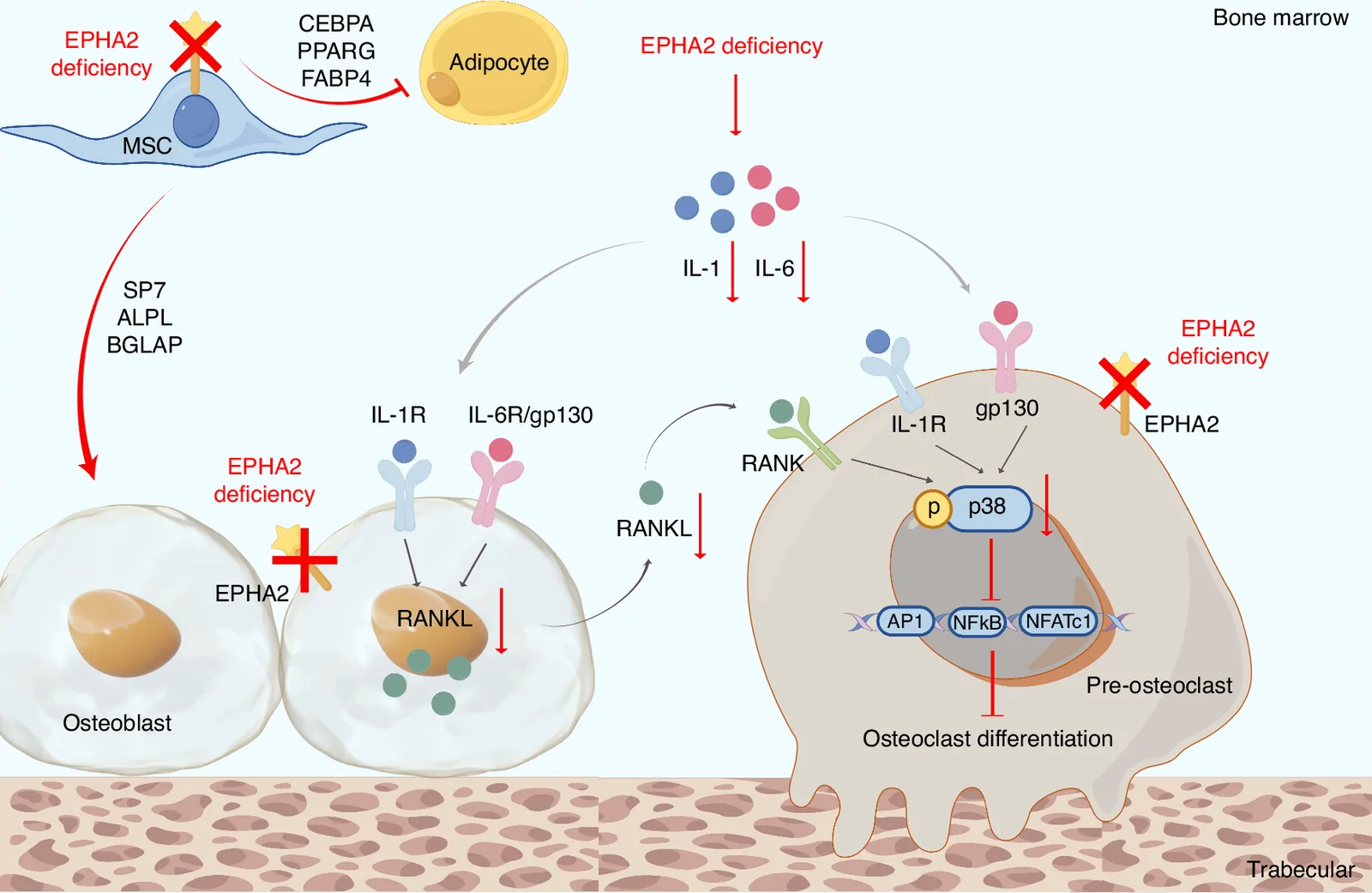
Proposed mechanistic diagram illustrating how EPHA2 loss-of-function mutations cause dysosteosclerosis. Left: Loss of EPHA2 in MSCs shifts lineage commitment toward osteogenesis (SP7/ALPL/BGLAP ↑ ; adipogenesis markers ↓ ). Middle: EPHA2 deficiency reduces IL-1β and IL-6 levels in bone, thereby attenuating IL-1β → IL1R and IL-6 → IL-6R/gp130 signaling and leading to decreased RANKL expression and secretion. Right: In pre-osteoclasts, reductions in bone IL-1β, IL-6, and RANKL blunt juxtacrine/paracrine inputs via RANKL → RANK, IL-1β → IL1R, and IL-6 → gp130. This attenuates p38 MAPK signaling, thereby weakening induction of c-Fos and NFATc1. Consequently, expression of fusion/effector genes (*DC-STAMP*, *ACP5*, *CTSK*, *MMP9*) declines, osteoclast differentiation and resorptive activity are collectively suppressed

To investigate the molecular mechanisms underlying the *EPHA2* gene mutation in DOS, we conducted analyses from multiple perspectives, including cell models, GWAS analysis, iPSCs models, and mouse models. Initially, we validated the pathogenicity of the S330fs and T297M mutations, both of which reduced EPHA2 protein expression and function and classified as loss-of-function mutations. GWAS studies have also confirmed that SNPs in the *EPHA2* gene are associated with BMD in the UK population. PBMC-induced osteoclastogenesis assay and iPSC cell model findings suggest functional abnormalities in the patient’s osteoblasts and osteoclasts. This was further validated in *Epha2*^*−/−*^ models. Further investigation in mice suggests that the *EPHA2* gene regulates bone remodeling by enhancing MSC osteogenic differentiation and downregulating the p38-MAPK pathway, leading to decreased osteoclast differentiation, as shown in Fig. 7.

Previous cellular studies have shown that ephrin-A2–EPHA2 contact-dependent, bidirectional signaling modulates osteoblast–osteoclast coupling, promoting osteoclastogenesis while restraining osteoblast differentiation.^26–28^ A recent study nominated ephrin-A1–EPHA2 signaling as a fracture prevention pathway and reported reduced total-body BMD in *Epha2*^*−/−*^ mice.^25^ This differs from our finding of increased trabecular BMD and improved trabecular microarchitecture in *Epha2*^*−/−*^ mice. We note that the prior report primarily used DXA-derived total-body BMD, which reflects an areal measure influenced by both cortical and trabecular bone, whereas we focused on site-specific micro-CT outcomes, and differences in sex, age, genetic background, and knockout strategy may also contribute.

A key aspect of sclerosing bone dysplasias research is the identification of therapeutic targets for osteoporosis. For example, loss-of-function mutations in the *SOST* gene encoding sclerostin lead to Sclerosteosis (OMIM 269500), Van Buchem disease (OMIM 239100) and Craniodiaphyseal dysplasia (OMIM 122860), three severe high BMD diseases. So sclerostin antibodies, such as romosozumab, can be used for osteoporosis treatment. To explore the therapeutic potential for osteoporosis, we treated OVX mice with ALW-II-41-27, one of the EPHA2 inhibitors. Our findings demonstrate that the inhibition of EPHA2 activity attenuated bone loss in OVX mice through dual action: suppression of osteoclasts and promotion of osteoblasts. Furthermore, the femoral fracture model indicates that EPHA2 deletion can promote fracture healing, raising the possibility that modulating EPHA2 signaling could benefit osteoporosis patients by improving both bone mass and repair capacity. Given EPHA2’s role in lens homeostasis, future therapeutic development should prioritize bone-targeted to maximize skeletal efficacy while minimizing systemic exposure.

This study has several limitations. First, as it is based on a single patient and our functional work largely relies on complete EPHA2 deletion, the phenotypic spectrum and the extent to which the patient’s compound heterozygous variants are equivalent to full loss of function require further validation. Second, because we used a global *Epha2* knockout model and assessed mice within a limited age window, systemic confounders and long-term skeletal dynamics cannot be fully excluded; although fat mass was not significantly different at 10 months (Fig. S4c) and prior work reported reduced baseline body weight in B6129S6-*Epha2*^tm1Jrui^/J (EPHA2-deficient) mice.^38^

In summary, we report a case of DOS caused by compound heterozygous mutation in the *EPHA2* gene, specifically S330fs and T297M. This finding expands the genetic and phenotypic spectrum of genetic skeletal disorders. We subsequently validated that loss-of-function mutations in the *EPHA2* gene lead to DOS through dual mechanisms: the inhibition of osteoclasts differentiation and function and the promotion of osteogenesis. Consequently, we proposed and validated *EPHA2* as a viable therapeutic target for osteoporosis. These findings offer novel insights for the development of osteoporosis therapeutics.

## Materials and methods

### Subjects

The Chinese Han family with DOS was included in this study. Clinical information, imaging records, and laboratory findings were collected. Laboratory tests were performed using an automated Roche electrochemiluminescence system (Roche Diagnostic GmbH), and BMD were measured with a DXA (Prodigy Advance, GE Lunar Corporation).^39^ Genomic DNA was extracted as described previously.^40^ WES and Sanger sequencing were performed for molecular diagnosis.

This study was approved by the Ethics Committee of Shanghai Sixth People’s Hospital Affiliated to Shanghai Jiao Tong University School of Medicine. Each subject signed informed consent before participation.

### WES and Sanger sequencing

WES was performed on three members of the family and analyzed as described previously.^40^ Candidate variants were confirmed by Sanger sequencing. The primer sequences were designed using Primer3 (v0.4.0). PCR products were purified and sequenced with a DNA sequencing system (3730XL; ABI).

### Bioinformatics analysis

PolyPhen-2, PROVEAN, and Mutation Taster were used to assess the damaging effects of mutations in silico.

### Cell culture

HEK293T cells were purchased from Shanghai Cell Bank of the Chinese Academy of Sciences and plated in DMEM supplemented with 10% fetal bovine serum (FBS) and 1% penicillin/streptomycin (P/S). Human BMSCs were purchased from Oricell and cultured in Complete Medium (HUXMA-90011, Oricell).

PBMCs of patient and his father were obtained using Ficoll-Paque PLUS (17144002, Cytiva) and plated in 24-well plates at approximately 2 × 10^5^ cells/mL in αMEM supplemented with 10% FBS and 1% P/S with 50 ng/mL macrophage colony-stimulating factor (M-CSF; 315-02, Peprotech) and 100 ng/mL RANKL (315-11, Peprotech). Osteoclast differentiation was assessed by TRAP staining.

### Plasmids and lentiviruses

EPHA2-WT, EPHA2-T297M, EPHA2-S330fs, and empty-vector constructs (3 × FLAG) were purchased from Hanbio Biotech (Shanghai, China). These plasmids were transiently transfected into HEK293T cells using Lipomaster 3000 Transfection Reagent (TL301, Vazyme Biotech Co.,Ltd). and EPHA2 protein expression levels were assessed by western blot.

For stable expression, human BMSCs were plated at 1.5 × 10^5^ cells/well in 12-well dishes then infected with lentivirus (MOI = 80), selected with puromycin (2 μg/mL) for 2 days after 72 h, and induced toward osteogenesis in αMEM containing 10% FBS, ascorbic acid (50 μg/mL), β-glycerophosphate (10 mmol/L), and dexamethasone (10 nmol/L). ARS staining was performed after 28 days.

### Association analysis of *EPHA2* and BMD

As we did in previous studies,^41–43^ the genetic individual-level data from the UK biobank (Application 41376) was used for association study with BMD. The UK Biobank cohort includes approximately 500 000 participants aged 40–69 years, among whom 487 409 were genotyped with the UK Biobank Axiom or UK UKBiLEVE Array, and then imputed by the 1000 Genomes Project (Phase 3) reference panel.^44,45^ Ethics approval for the UK Biobank research was obtained from the North West Multicentre Research Ethical Committee, and all participants provided informed consent (original ethics committee approval number: 21/NW/0157). In this study, we extracted BMD traits measured by DXA from 11 anatomical sites (arm, total femur, femoral neck, head, legs, lumbar spine, pelvis, ribs, spine, trunk, and total body) as phenotypes. To minimize the population stratification bias, individuals of non-European ancestry and those who had a kinship with any participants were excluded. For quality control of genotype data, the variants were excluded if the minor allele frequency (MAF) < 0.01, imputation info score < 0.3, missing genotype rates > 0.05, and *P*-value for Hardy–Weinberg equilibrium test < 1 × 10^−^^6^. After the quality control, approximately 30 000 participations remained for BMD GWAS analysis.

To identify the *EPHA2* variants associated with BMD, GWAS analysis was conducted for BMD traits at 11 skeletal sites. BMD measurements (g/cm²) were stratified by sex and adjusted for relevant covariates including age, age^2^ body weight, menopausal status (females only), and the first five principal components using linear regression. The standardized residuals (mean = 0 and sd = 1) were calculated separately for males and females and used as the outcome variables in GWAS. The associations between *EPHA2* variants and standardized-BMD at 11 skeletal sites were analyzed in PLINK. Summary statistics from male and female analyses were then combined via inverse variance weighted fixed effects meta-analysis in METAL.^46^ The lead SNP of novel loci with *P*-value from sex-stratified GWASs less than 0.05 were considered to be replicated.

### iPSCs

iPSCs were obtained from PBMCs of both the patient and the patient’s parents as described previously,^47^ cultured under feeder-free conditions, and differentiated into MPCs using STEMdiff™ Mesenchymal Progenitor Kit (05240, STEMCELL). MPCs were induced toward osteogenic, adipogenic, or chondrogenic lineages using standard protocols. Differentiation was evaluated by ARS staining (day 28, osteogenesis), Oil Red O staining (day 21, adipogenesis), and alcian blue staining (day 21, chondrogenesis).

### Generation of *Epha2*^*−/−*^ knockout mice

*Epha2*^*−/−*^ mouse model was designed and generated from GemPharmatech Co., Ltd, using CRISPR-Cas9 technology as described previously.^48^ All animal experiments were approved by the Ethics Committee of the Shanghai Sixth People’s Hospital Affiliated to Shanghai Jiao Tong University School of Medicine.

### Micro-CT analysis

Micro-CT analysis was performed using a Skyscan 1176 system (Bruker, Kontich, Belgium). The reconstruction and trabecular bone parameters were analyzed using the Skyscan, NRecon, Data Viewer, CTAn, CTvox, and Batman software.

### Serum bone turnover markers

Serum of 12-month-old male *Epha2*^*−/−*^ mice and WT mice was collected and stored at -80 °C. Expression levels of CTX and OC were estimated using mouse ELISA Kit (BYHS101049 and BYGR507986, BYabscience), according to the manufacturer’s protocols.

### In vitro osteogenic and adipogenic differentiation of BMSCs

Bone marrow cells were harvested and cultured in αMEM supplemented with 10% FBS and 1% P/S with regular media changes to obtain mice BMSCs. Then BMSCs were induced toward osteogenic and adipogenic differentiation. ALP staining (day 7), ARS staining (day 28), and Oil Red O staining (day 7) were performed after fixation.

### CFU-F assay

1 × 10^6^ bone marrow cells were seeded in a six-well plate in αMEM supplemented with 10% FBS and 1% P/S with regular media changes. After 12 days of culture, cells were fixed with 4% PFA and stained with crystal violet Staining Solution.

### Calcein double labeling

Intraperitoneal injection of 200 μL of 2 mg/mL calcein into 10-month-old *Epha2*^*−/−*^ and WT mice, followed by a second intraperitoneal injection 7 days later. Harvest femurs 24 h post-second injection. Femurs fixed with 4% PFA and then embedded in methylmethacrylate.

### In Vitro Differentiation of Osteoclast

Osteoclast differentiation of mice was described previously.^40^ BMMs of *Epha2*^*−/−*^ and WT mice were allowed to adhere in the presence of M-CSF (50 ng/mL), after which cultures were maintained with or without pamapimod (64 nmol/L, HY-10405, MCE). At the start of induction, cultures received IL-1β (100 ng/mL; 211-11B, PeproTech) and/or IL-6 (100 ng/mL; 216-16, PeproTech) in four conditions: RANKL alone, IL-1β + RANKL, IL-6 + RANKL, or IL-1β + IL-6 + RANKL; WT were included only under RANKL alone as a reference control. Osteoclast differentiation was assessed by TRAP staining and resorption pit staining after 5 days of induction. Detailed procedures for the transwell co-culture assay are provided in the Supplementary Methods.

### Bone histology and immunohistochemistry staining

Following decalcification, femurs were paraffin-embedded for TRAP staining and immunohistochemistry staining. Non-decalcified femurs were embedded in methylmethacrylate for masson staining. Immunohistochemistry staining was performed as described previously.^48^ The sections were incubated with rabbit anti-Osteocalcin antibodies (1:500; ab93876, Abcam) or anti-Sp7/Osterix antibodies (1:500; ab209484, Abcam).

### Western blot analysis

Protein extraction and western blot were performed as described previously.^48^ Each sample was probed for p38 MAPK (1:1 000 dilution, 9212, CST), phospho-p38 MAPK (Thr180/Tyr182) (1:1 000 dilution, 9211, CST) and β-tubulin (1:1 000 dilution, T40002, Abmart).

### Real-time PCR analysis

The experimental methods were performed as previously described.^48^ Sequences of the primers are listed in Table S4.

### RNA sequencing and analysis

We collected osteoclasts of *Epha2*^*−/−*^ mice and WT mice and obtained RNA for transcriptome sequencing (fold change > 2.0 and *P* < 0.05). The data were analyzed on the free online platform of Novogene (https://magic.novogene.com).

### Femoral fracture model

To investigate the impact of the *EPHA2* gene on fracture healing, we established the femoral fracture model using 6-month-old *Epha2*^*−/−*^ and WT mice. Following anesthesia, intramedullary nails were implanted in the femurs. A preload of 1.0 N was applied, and a controlled fracture was induced at a constant speed of 0.2 mm/s, resulting in a middiaphyseal femoral fracture. X-ray assessments were performed at 14, 21, and 28 days after the fracture, with micro-CT scans conducted at 28 days. X-rays were performed as previously described.^48^

### OVX mice model

To investigate whether EPHA2 can serve as a new therapeutic target for osteoporosis, we established the OVX mice models and administered EPHA2 inhibitor treatment. The experimental plan is outlined as follows. Following baseline micro-CT scans of all 2-month-old female mice, bilateral OVX was performed on the OVX groups, while the ovaries were raised but not resected on the sham group. One week after OVX, for the treated groups, 50, 100, 200, and 400 μg/kg ALW-II-41-27 (HY-18007, MCE), the EPHA2 tyrosine kinase inhibitor, was intraperitoneally injected twice daily for 14 days. The same volume of saline was given to the untreated groups. All mice underwent a second micro-CT scan one week after the completion of treatment. Then, all mice were sacrificed, and femurs were obtained for histological staining.

### Figure preparation

Immunohistochemistry was quantified in ImageJ. Histograms were generated in GraphPad Prism. Primary figures were assembled in Adobe Illustrator, and mechanism diagrams were created with Figdraw.

### Statistical analysis

All the experiments were biologically repeated at least three times, and the results are reported as means ± SEM. Two-tailed Student’s *t*-tests were performed for two groups. Differences were considered statistically significant at *P* < 0.05. Unless otherwise specified, significance was defined as *P < 0.05, **P < 0.01, ***P < 0.001, ****P < 0.000 1; ns, not significant.

## Supplementary information

Supplementary Information

Supplementary Table 3

## Data Availability

The datasets generated and analyzed during the current study are available from the corresponding author upon reasonable request. Individual-level human genomic and clinical data are not publicly available due to privacy restrictions. UK Biobank data are available through application to the UK Biobank (Application 41376).
