# Supplementary material for: Bi-allelic EPHA2 mutations cause dysosteosclerosis by inhibiting osteoclastogenesis and enhancing osteoblastogenesis
Source: Bone Res. 2026 Oct 12;14:104. doi: 10.1038/s41413-026-00565-x (PMC13658827; doi:10.1038/s41413-026-00565-x)
Supplement: Supplementary file 1 [file 41413_2026_565_MOESM1_ESM.docx]

**Bi-allelic *EPHA2* Mutations Cause Dysosteosclerosis by Inhibiting Osteoclastogenesis and Enhancing Osteoblastogenesis**

Xi Chen *et al*.

* Correspondence: Hou-Feng Zheng ([houf.zheng@suda.edu.cn](mailto:houf.zheng@suda.edu.cn)); Weiwei Hu ([huweiwei1980@163.com](mailto:huweiwei1980@163.com)) and Zhenlin Zhang ([zhangzl@sjtu.edu.cn](mailto:zhangzl@sjtu.edu.cn))

This supplementary information file includes:

Supplementary Figures 1-7

Supplementary Tables 1-2 and 4

Supplementary Methods

**Supplementary Figure 1 Slit-lamp examination of the family members.** Ⅰ-1, patient’s father; Ⅰ-2, patient’s mother; Ⅱ-1, the patient.

**Supplementary Figure 2 Characterization of established iPSCs.** (a) Karyotyping results of iPSCs; (b) Photomicrograph of cell morphology; (c) qRT-PCR results of key pluripotent genes; (d) Validation of *EPHA2* gene mutation sites in iPSCs.

**Supplementary Figure 3 Regional association plots of flanking 250kb region around the *EHPA2* gene region, based CPASSOC results of 11 DXA-derived BMD.** The x-axis denotes the physical position of each genetic variant on the chromosome specified, whereas the y-axis indicates the evidence of association, which was shown as -log_10_(*P*-value).

Abbreviations: BMD, bone mineral density; CPASSOC: Cross-phenotype association analysis; DXA, dual-energy X-ray absorptiometry.

**Supplementary Figure 4 Validation of the *Epha2^-/-^* model and body composition analysis.** (a) Genotyping of WT (*Epha2^+/+^*), HE (*Epha2^+/-^*), and HO (*Epha2^-/-^*) mice; NC, negative control; (b) EPHA2 protein levels in bone, spleen, and liver tissues from *Epha2^-/-^* and WT mice (n=3); (c) Body composition analysis of 10-month-old male *Epha2^-/-^* and WT mice (n=3).

**Supplementary Figure 5 Micro-CT analysis of *Epha2^-/-^* mice across multiple ages.** (a) 3-month-old male *Epha2^-/-^* mice bone parameter analysis (n=4); (b) 6-month-old male *Epha2^-/-^* mice bone parameter analysis (n=3); (c) 14-month-old male *Epha2^-/-^* mice bone parameter analysis (n=3); (d) 12-month-old female *Epha2^-/-^* mice bone parameter analysis (*Epha2^-/-^* n=4; WT n=6).

**Supplementary Figure 6 Masson staining analyses and transwell co-culture assay results in *Epha2^-/-^*** **mice.** (a) Masson staining of undecalcified femoral tissue sections of 14-month-old *Epha2^-/-^* mice and WT mice (n=3); (b) *Epha2^-/-^* BMMs were seeded in the lower wells, and WT or *Epha2^-/-^* BMSCs or osteoblast lineage cells (OLCs) were seeded in the upper inserts. Osteoclastogenesis was induced with M-CSF and RANKL (n=3).

**Supplementary Figure 7 EPHA2 deficiency accelerates fracture repair.** (a) X-ray results of 6-month-old *Epha2^-/-^* mice and WT mice at 14, 21, and 28 days after bone fracture; (b) Micro-CT 3D reconstruction of 6-month-old male *Epha2^-/-^* mice and WT mice at 28 days post-fracture; (c) Micro-CT analysis of bone microstructure at 28 days post-fracture (n=5).

Supplementary Table 1 Refractive examination results

|  | | SPH | CYL | AXIS | VA | ADD |
| --- | --- | --- | --- | --- | --- | --- |
| Mother  (I-2) | OD | -4.75 | -0.5 | 165 | 0.8 | +1.00 |
|  | OS | -2.25 | -0.25 | 15 | 0.8- | +1.00 |
| Father  (I-1) | OD | +0.00 | - | - | 1.0 | +0.5 |
|  | OS | +0.00 | - | - | 1.0 | +0.5 |
| Patient  (Ⅱ-1) | OD | +0.00 | -2.75 | 180 | 1.0 | - |
|  | OS | +0.00 | -2.75 | 170 | 1.0 | - |

Abbreviations: SPH, Sphere; CYL, Cylinder; ADD, Addition; VA, Visual Acuity; OD, Oculus Dexter; OS, Oculus Sinister.

Supplementary Table 2 bioinformatic analysis of *EPHA2* mutations

| base on db cDNA | base on db protein | MutationTaster | PolyPhen-2 | SIFT Score |
| --- | --- | --- | --- | --- |
| c.890C>T | p.Thr297Met  (T297M) | disease causing | probably damaging | damaging |
| c.988dupT | p.Ser330PhefsX51  (S330fs) | disease causing | possibly damaging | damaging |
|  |  |  |  |  |

Supplementary Table 4 Primer sequences for quantitative real time PCR

|  | Forward (5'-3') | Reverse (5'-3') |
| --- | --- | --- |
| *β-actin* | AACAGTCCGCCTAGAAGCAC | CGTTGACATCCGTAAAGACC |
| *Alpl* | GCCATTGGCACCTGCCTTAC | AGCTCCAGGCATATTTCAGTGTC |
| *Bglap* | CTGACCTCACAGATCCCAAGC | TGGTCTGATAGCTCGTCACAAG |
| *Sp7* | CCTCTGCGGGACTCAACAAC | AGCCCATTAGTGCTTGTAAAGG |
| *Tnfrs11b* | AGCTGCTGAAGCTGTGGAA | GGTTCGAGTGGCCGAGAT |
| *Tnfsf11* | GCAGAAGGAACTGCAACACA | GATGGTGAGGTGTGCAAATG |
| *Ctsk* | CTTCCAATACGTGCAGCAGA | TCTTCAGGGCTTTCTCGTTC |
| *Dcstamp* | TCCTCCATGAACAAACAGTTCCAA | AGACGTGGTTTAGGAATGCAGCTC |
| *Mmp9* | AGACGACATAGACGGCATC | TGCTGTCGGCTGTGGTTC |
| *Nfatc1* | CCGTTGCTTCCAAAAATAACA | TGTGGGATGTGAACTCGGAA |
| *Tnfrs11a* | TCTGCAGCTCTTCCATGACACT | GAAGAGGAGCAGAACGATGAGACT |
| *Acp5* | TCCGTGCTCGGCGATGGACCAGA | CTGGAGTGCACGATGCCAGCGACA |
| *Fos* | CCAGTCAAGAGCATCAGCAA | AAGTAGTGCAGCCCGGAGTA |
| *Il1b* | GAAGTTGACGGACCCCAAAAG | TGCGAGATTTGAAGCTGGATG |
| *Il6* | GTTGCCTTCTTGGGACTGATG | GCCATTGCACAACTCTTTTCTC |
| *Cebpa* | TGGACAAGAACAGCAACGAG | TCACTGGTCAACTCCAGCAC |
| *Pparg* | GGAAAGACAACGGACAAATCA | TACGGATCGAAACTGGCAC |
| *Fabp4* | GATGAAATCACCGCAGACGACA | ATTGTGGTCGACTTTCCATCCC |
| *β-ACTIN* | CATGTACGTTGCTATCCAGGC | CTCCTTAATGTCACGCACGAT |
| *SOX2* | GGTTACCTCTTCCTCCCACTCCAG | TCCCCTTCTCCAGTTCGCAGTC |
| *POU5F1* | GAGTGTGGTTCTGTAACCGGC | GCCAGAGGAAAGGACACTGGT |
| *NANOG* | GCCCTGATTCTTCCACCAGTC | GCTGGGTGGAAGAGAACACAG |
| *EPHA2* | TGGCTCACACACCCGTATG | GTCGCCAGACATCACGTTG |
| *FOS* | CCGGGGATAGCCTCTCTTACT | CCAGGTCCGTGCAGAAGTC |
| *NFATC1* | CACCGCATCACAGGGAAGAC | GCACAGTCAATGACGGCTC |
| *COL10* | ATGCTGCCACAAATACCCTTT | GGTAGTGGGCCTTTTATGCCT |
| *MMP13* | ACTGAGAGGCTCCGAGAAATG | GAACCCCGCATCTTGGCTT |
| *FABP4* | ACTGGGCCAGGAATTTGACG | CTCGTGGAAGTGACGCCTT |
| *PPARG* | GGGATCAGCTCCGTGGATCT | TGCACTTTGGTACTCTTGAAGTT |
| *ALPL* | ACCACCACGAGAGTGAACCA | CGTTGTCTGAGTACCAGTCCC |
| *BGLAP* | CACTCCTCGCCCTATTGGC | CCCTCCTGCTTGGACACAAAG |

Supplementary Methods

Transwell co-culture assay was performed to evaluate osteoblast-lineage support of osteoclastogenesis. Osteoblast lineage cells (OLCs) were isolated from mouse cortical bone by enzymatic digestion with type II collagenase (3 mg/mL), dispase II (4 mg/mL), and DNase I (1 U/mL) for 30 min at 37 °C in a water bath. Cells were plated in αMEM supplemented with 10% FBS and 1% P/S, and adherent cells were collected and used as OLCs. WT or *Epha2^-/-^* OLCs (or BMSCs) were seeded into transwell inserts at 2×10^4^ cells/well in αMEM supplemented with 10% FBS and 1% P/S (upper chamber). After attachment, *Epha2^-/-^* BMMs were seeded into the lower wells at 5×10^5^ cells/well. The upper chamber contained αMEM supplemented with 10% FBS and 1% P/S, whereas the lower chamber contained α-MEM supplemented with 10% FBS and 1% P/S, with M-CSF (50 ng/mL) and RANKL (100 ng/mL) to induce osteoclast differentiation. Medium was refreshed on day 3, and osteoclastogenesis was assessed by TRAP staining on day 4.
